# IMU-Based Gait Recognition Using Convolutional Neural Networks and Multi-Sensor Fusion

**DOI:** 10.3390/s17122735

**Published:** 2017-11-27

**Authors:** Omid Dehzangi, Mojtaba Taherisadr, Raghvendar ChangalVala

**Affiliations:** Computer and Information Science Department, University of Michigan-Dearborn, Dearborn, MI 48128, USA; dehzangi@umich.edu (O.D.); rchangal@umich.edu (R.C.)

**Keywords:** gait identification, inertial motion analysis, spectro-temporal representation, deep convolutional neural network, multi-sensor fusion, error minimization

## Abstract

The wide spread usage of wearable sensors such as in smart watches has provided continuous access to valuable user generated data such as human motion that could be used to identify an individual based on his/her motion patterns such as, gait. Several methods have been suggested to extract various heuristic and high-level features from gait motion data to identify discriminative gait signatures and distinguish the target individual from others. However, the manual and hand crafted feature extraction is error prone and subjective. Furthermore, the motion data collected from inertial sensors have complex structure and the detachment between manual feature extraction module and the predictive learning models might limit the generalization capabilities. In this paper, we propose a novel approach for human gait identification using time-frequency (TF) expansion of human gait cycles in order to capture joint 2 dimensional (2D) spectral and temporal patterns of gait cycles. Then, we design a deep convolutional neural network (DCNN) learning to extract discriminative features from the 2D expanded gait cycles and jointly optimize the identification model and the spectro-temporal features in a discriminative fashion. We collect raw motion data from five inertial sensors placed at the chest, lower-back, right hand wrist, right knee, and right ankle of each human subject synchronously in order to investigate the impact of sensor location on the gait identification performance. We then present two methods for early (input level) and late (decision score level) multi-sensor fusion to improve the gait identification generalization performance. We specifically propose the minimum error score fusion (MESF) method that discriminatively learns the linear fusion weights of individual DCNN scores at the decision level by minimizing the error rate on the training data in an iterative manner. 10 subjects participated in this study and hence, the problem is a 10-class identification task. Based on our experimental results, 91% subject identification accuracy was achieved using the best individual IMU and 2DTF-DCNN. We then investigated our proposed early and late sensor fusion approaches, which improved the gait identification accuracy of the system to 93.36% and 97.06%, respectively.

## 1. Introduction

Gait refers to the manner of stepping or walking of an individual. Human gait analysis research dates to the 1960s [1] when it was used for medical purposes for early diagnosis of various disorders such as neurological disorders such as Cerebral Palsy, Parkinson’s or Rett syndrome [2], musculoskeletal disorders such as spinalstenosis [3], and disorders caused by aging, affecting large percentage of population [4].

Reliable monitoring of gait characteristics over time was shown to be helpful in early diagnosis of diseases and their complexities. More recently, gait analysis has been employed to identify an individual from others. Unlike iris, face, fingerprint, palm veins, or other biometric identifiers, gait pattern can be collected at a distance unobtrusively [5]. In addition, recent medical studies illustrated that there are 24 various components to human gait and that gait can be unique if all movements are considered [6]. As a result, gait has the potential to be used for biometric identification. It is particularly significant as gait patterns are naturally generated and can be seamlessly used for authentication under smart and connected platforms e.g., keyless smart vehicle/home entry [7], health monitoring [8], etc.

In recent years, there has been much effort on employing wearable devices for activity recognition [9], activity level estimation [10], joint angle estimation [11], activity-based prompting [12], and sports training [13]. Recently, gait identification using wearable motion sensors has become an active research topic because of the widespread installation of sensors for measuring movements in smartphones, fitness trackers, and smartwatches [7,14]. Most of the wearable motion sensors use Micro Electro Mechanical Systems (MEMS) based inertial sensors. These inertial sensors (accelerometers, gyroscopes) are one of the most important members of MEMS family and are combined together as inertial measurement units (IMU). Most modern accelerometers are electromechanical devices that measure acceleration forces in one, two, or three orthogonal axes. Gyroscope sensors are devices that measure angular velocity in three directions. Due to their small-size, portability, and high processing power, IMUs are widely used for complex motion analysis. Hence, gait recognition using wearable IMUs has become an efficient Privacy Enhancing Technology (PET) [15].

Advent of MEMS-based accelerometers and gyroscopes and wireless interfaces such as Bluetooth and Wi-Fi have made the measurement setup for gait analysis data collection non-intrusive and ubiquitous. IMUs have become a significant part of ubiquitous smart devices and therefore integration of inertial sensors in smart devices has become a common practice. There is a mass of people using smart devices on a daily basis. With the latest achievements in the field of pervasive computing, limitations of inertial sensors such as cost, storage, and computational power were overcome to a great extent [16]. Therefore, inertial sensors are not only restricted to simple tasks such as tilt estimation but also for complex tasks such as advanced motion analysis, and activity recognition [14]. They have also been evaluated in medical applications, such as analysis of patient’s health based on gait abnormalities [17], fall detection [18]. Although the IMU-based wearables have enabled pervasive motion and gait analysis, there are some intrinsic challenges with those devices. Since, the wearable device is always worn casually, relative orientation between the sensors and the subject body cannot be fixed over different sessions of data acquisition [19]. As the coordinate system used by sensors is defined relative to the frame of the device, small orientation changes of sensor installation may make measurements quite different [19]. The issue of ensuring orientation invariance in extracting gait features has been a matter of concern in many studies. In [20], the authors introduced an orientation invariant measure to alleviate the orientation dependency issue. In this work, we also employ an orientation invariant resulting measures of motion as the input signals.

A large number of research studies have been conducted on developing gait recognition systems using inertial sensors. As a pioneer study in this field, in [21], a triaxial accelerometer was used and fixed on a belt to keep the relative position between the subject’s body and sensors unchanged. In order to detect the gait cycles, they have applied a peak detection method. Then, they implemented a template matching process to identify their subjects from their gaits. Due to the less contribution of axis *Y*, authors just used *X* and *Z* axes. Similarity-based measures and machine learning are two frequently used approaches for gait identification in the recent literature. Similarity-based measures such as Tanimoto distance [22], dynamic time warping metrics (DTW) [23], and Euclidean distance [24] have been used in the recent studies. Similarity-based approaches are dependent on selecting representative gait patterns (commonly by an expert and manually) and requires storing them for all subjects in order to compare them with the search population, which, in turn, results in lower efficiency in storage and computation. Machine learning techniques are commonly designed in two major modules after pre-processing of the motion data: (1) feature extraction from the input signal in short windows of the streaming data; and (2) model training to generate a predictive model fed by the data at the feature space. Various modeling algorithms such as Bayesian network classifier [25], hidden Markov model classifier [24], support vector machines, and decision trees [25] have been used in gait recognition applications. The performance of such systems are highly dependent on the extracted features and their resulting hypothesis class, which is the set of possible predictors with the fixed set of features. The impact of noise interference and particularly motion artifacts on complex sensor data makes the task of extracting relevant and robust features very challenging. Commonly, feature extraction is undertaken manually and via handcraft effort for a specific application [15]. However, extracting manual and hand crafted features for machine learning based systems is cumbersome, subjective, and is prone to biases due to the complexity of sensor data collected from IMUs [26,27]. Manual feature extraction is heuristic and can result in poor expressivity of the feature set (i.e., the set of possible predictors with a fixed set of features may not be good enough). Therefore, the best model given the manual features might generate poor accuracy compared to an optimal performance given the desired representative feature subspace. Another important reason for poor expressivity of commonly used machine learning-based methods can be the detachment of feature extraction and the predictive model training. In this way, important information that might be crucial for high performance predictive modeling, can be neglected in the process of feature extraction.

In this paper, we propose a gait recognition framework and investigate the ability to extract time-invariant signature motion patterns within a gait cycle of each subject to identify him/her from others. We first exploit the information provided by expanding the motion signals recorded from various IMUs worn by the participants to 2D spectro-temporal space via time-frequency (TF) analysis. Due to the non-stationarity of the motion signals, TF and instantaneous frequency (IF)-based methods accommodate the temporal variations in the gait patterns during a gait cycle segment. However, there are two major issues in order to extract relevant descriptors from the 2D TFs: (a) efficient selection of relevant features from the 2D spectro-temporal expanded space might not be feasible due to high dimensionality of the space; and (b) selection of a reliable predictive model given the 2D TFs is a difficult task due to the high dimensionality of the input space and shallow models might face the challenge of curse of dimensionality as discussed in [28]. The authors in [28] discussed that placing decision hyperplanes directly the high dimension space space (the high resolution 2D TF in this work) might raise the risk of curse of dimensionality and hurt the generalization ability of the learnt predictive model. They illustrated that incorporating hierarchical locality using deep learning structures can be sufficient to avoid the curse of dimensionality [28]. Therefore, in this paper, we design a deep convolutional neural network (DCNN) model that is trained for each of the sensor nodes (i.e., five inertial sensors) and the modalities (i.e., accelerometer and Gyroscope readings) to extract individual signature patterns from the 2D expanded gait cycles and optimize the predictive identification model at the same time in a discriminative fashion. The best individual DCNN performance reaches 91% identification accuracy. In order to aggregate the complementary discriminative information from all sensors, we then investigate multi-sensor early and late fusion with the aim of improving the gait identification performance. We achieve the average accuracy of 93.36% via early fusion by augmenting multi-sensor gait cycles at the input level. In late fusion, a discriminative performance measure is introduced that directly relates the performance of the fusion of individual sensor DCNN models to the fusion weight parameters. Using the introduced measure, we propose the minimum error score fusion (MESF) learning method that discriminatively optimizes the linear fusion weights of DCNN scores at the score level by minimizing the error rate on the training data in an iterative manner. The average gait identification accuracy of 97.06% is achieved by applying our proposed MESF method on the DCNN decision scores.

## 2. Method

**Figure 1:**
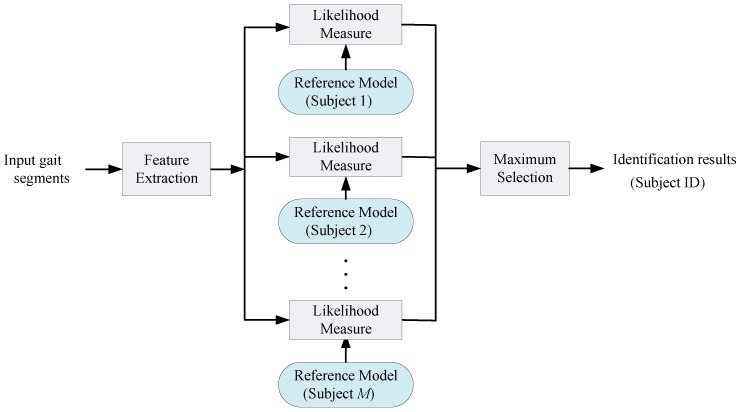
Human gait identification task.

We aim to automatically identify a target subject given their gait information. Assuming *M* target subjects, given an unknown gait segment, a gait identification system gives the corresponding subject identity, φ, where $\varphi \in \left\{1,\cdots ,M\right\}$. Figure 1 illustrates the structure of the gait identification task. As shown in Figure 1, gait segments are processed and relevant features are extracted from them either manually or automatically. Then, a set of reference models, learned in the training phase using a set of training data, is employed to classify the input gait segments to one of the *M* subjects. Each model, φ, generates a likelihood score of an input gait segment belonging to the target subject φ. The aim is to identify each subject based on their individual gait characteristics given that all the subjects are performing the same activity i.e., gait. In order to achieved this goal in this paper, we capture and identify visual high level spectro-temporal features in an isolated gait cycle in a discriminative fashion using DCNN and multi-sensor fusion.

**Figure 2:**
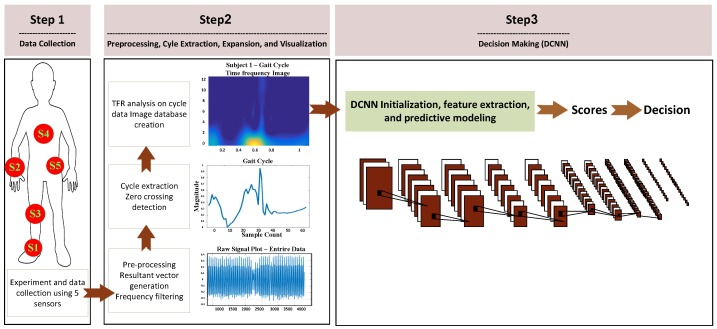
The overview of our proposed system for Human Gait Identification.

The overview of the proposed gait identification system is depicted in Figure 2. Raw motion data is collected from five inertial sensors worn by a population of subjects. Then, gait cycles are extracted and transformed to 2D TF space. The high-level one-vs-rest subject discriminative features of the expanded gait cycles are captured through the 10-layer hierarchies of the DCNN and predictive model training is conducted jointly using the last layers of the DCNN network to perform the human identification task. We then combine the individual sensor systems via performing multi-sensor early and late fusion.

### 2.1. Experimental Setup and Data Collection

A total of 10 subjects participated in the experimental procedure. In this way, we present a solution for a 10-class problem as a proof of concept of identifying a target person among overall 10 subject population (with 10% expected accuracy of random guess). A set of five inertial sensors were placed at various locations including chest, right wrist, right knee, right ankle and lower back in order to conduct a gait identification performance comparison between different sensor locations and improving the overall performance via multi-sensor fusion. Table 1 provides the detailed characteristics of the sensors [29,30]. The motion sensor system employed for this study was Shimmer sensor platform, which is a wearable senor platform with wireless interface. It houses both the accelerometer and gyroscope inertial sensors. The data collection sessions were synchronized across all the sensors and labeled using our in-lab designed Android application developed at the Wearable Sensing and Signal Processing (WSSP) laboratory, University of Michigan, Dearborn. Each subject was asked to walk the same route from a specific point to another outside the building.

### 2.2. Preprocessing

**Figure 3:**
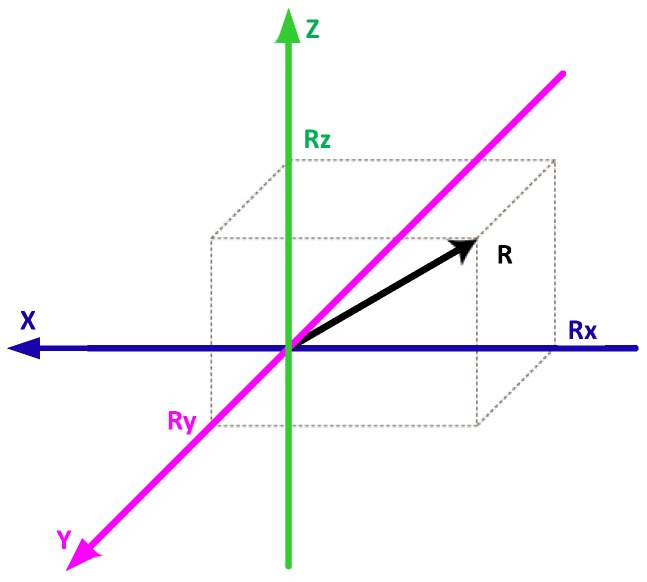
Resulting Factor Extraction. *R* indicates resulting factor.

The raw accelerometer and gyroscope data ($R_{x}$, $R_{y}$ and $R_{z}$ vectors) collected during the experiment is contaminated with various noise factors such as motion artifacts, step impacts, sensor orientation and location related noises along with the necessary gait information. To alleviate the orientation related biases, resultant vectors of the triaxial sensor data (i.e., accelerometer and gyroscope recorded data) is computed using Equation (1). Figure 3 illustrates a visualization of resulting factor extraction, which is an orientation invariant measure of overall acceleration and angular velocity of each IMU.

(1) $$Mag\left(t\right)=R=\sqrt{{R_{x}}^{2}\left(t\right)+{R_{y}}^{2}\left(t\right)+{R_{z}}^{2}\left(t\right)}$$

Since typical gait data for normal walking has frequency components in the range of 0.5 to 3.5 Hz, a 10-th order Butterworth bandpass filter was used to extract the required frequency components from the resultant vectors of the IMUs. For Gyroscope data, we assume the first estimated value of direction vectors to be the same as the direction vectors measured by the accelerometer:

(2) $$\begin{array}{c} R_{x}Est\left(0\right)=R_{x}Acc\left(0\right)\\ R_{y}Est\left(0\right)=R_{y}Acc\left(0\right)\\ R_{z}Est\left(0\right)=R_{z}Acc\left(0\right) \end{array}$$

In our algorithm, we assume the value of the accelerometer, when the sensor device is at rest (a first few seconds recordings before subjects started the gait paradigm), to be zero.

### 2.3. Gait Cycle Extraction

Since the data collection through all the sensors is time synchronized, we extracted the gait cycles from the sensor#1 (i.e., the ankle sensor) and used the same markers for other sensors. The cycle extraction process implements amplitude check and zero crossing check to extract noise free gait cycle data.

To approximate the gait cycle frequency, the resultant vectors of accelerometer data from sensor#1 $R_{Acc}$ are passed through a band pass filter of rage 0.5–1.5 Hz. We conduct a more aggressive band pass filtering only for the purpose of cycle extraction. This eliminates the interference of any high frequency components while determining the gait cycle frequency. The frequency range of 0.5–1.5 Hz is chosen to include the average gait frequency, which is 1 Hz. Frequency Analysis consists of finding the energy distribution as a function of the frequency index ω. Therefore, it is necessary to transform the signal to the frequency domain by means of the Fourier transformation:

(3) $$X\left(\omega \right)=\int x\left(t\right)e^{-jt\omega }dt$$

where x(t) is the time domain signal. A Fourier transform is performed on the resultant data and the dominant frequency component within the range of 0.5 to 1.5 Hz is selected as the gait cycle frequency of that subject $f_{cycle}$.

In order to remove irregular gait cycles, the $R_{Acc}$ is checked against the amplitude threshold of a signal 0.05. If there are samples that have values below the threshold, those samples are neglected and samples with an amplitude above the threshold are collected into small local windows. Gait cycles are extracted from these local windows *(LW)* following the process shown in the Algorithm 1.

**Algorithm 1:** Gait Cycle Extraction Process $S_{cycleStart}$ = 0           *while* ($LW_{end}$ < length(LW))                     $S_{cycleEndTemp}$ = $S_{cycleStart}$ + $S_{cycleOffset}$                     $S_{cycleEnd}$ = $S_{cycleEndTemp}$ ± $S_{zeroCrossing}$                     $S_{cycleStart}$ = $S_{cycleEnd}$                     $LW_{end}$ = $S_{cycleStart}$           *end*

To synchronize the cycles, zero crossing of the $R_{Acc}$ is taken as a reference point and the samples between a given zero crossing and the subsequent zero crossing at $S_{cycleOffset}$ samples away from previous zero crossing is considered to be a gait cycle. $S_{cycleOffset}$ is calculated using Equation (4), where $f_{s}$ is the data sampling frequency 50 Hz in our empirical investigations.

(4) $$S_{cycleOffset}=\frac{f_{s}}{f_{cycle}}$$

The end sample of this cycle $S_{cycleEndTemp}$ is approximated initially based on the $f_{cycle}$ but a more precise $S_{cycleEnd}$ is calculated based on finding a zero-crossing sample near the initial approximation. The local window indices of the start and end of cycle data $S_{cycleStart}$ and $S_{cycleEnd}$ are then mapped to the global window of $R_{Acc}$.

**Figure 4:**
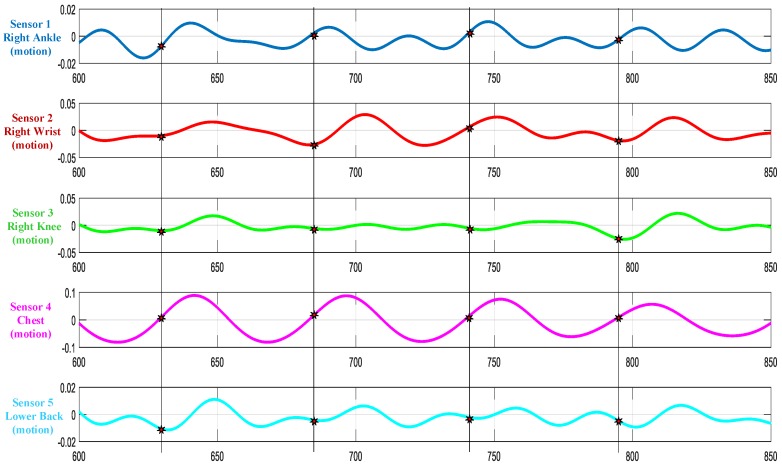
Extracted cycle data sample plot for subject 1 across all the sensors.

Cycle data collected across all subjects and sensors were observed for data consistency, i.e., the number of gait cycles extracted per sensor. Considering this number as a measure of data integrity, the ankle-based accelerometer (sensor#1) captured nearly all of the gait cycles performed by different subjects consistently. Hence, it was selected as the reference sensor with discriminative features to represent the gait cycle. Gait cycles are extracted from other sensors based on the indices generated from the sensor#1 $R_{Acc}$. Figure 4 shows the gait cycle extracted from Subject#1 for all the five sensors.

### 2.4. Time Frequency Representation

One way to represent and describe a multi-component and non-stationary signal simultaneously in frequency and time space is considering its instantaneous frequency (IF). To analyze a multi-component signal, an IF rule can be assigned to all components of the signal. Several IF estimation methods have been considered for multi-component signals in recent literatures [31]. These methods first characterize and extract components of the signal and then conduct an IF estimation procedure. Implementation of a multi-component IF estimation approach includes two major steps as follows:

1. Applying time frequency transformation.
  First step is mapping the input signal to the time and frequency space by applying an appropriate time-frequency distribution (TFD). TFD method can be chosen by considering the characteristics of the input signal.
2. Choosing a proper method for estimating IF.
  Methods for estimating IF consider the TFD space (G(T,F)) as a two dimensional representation, which its coordinates are row (time) and column (frequency) of the *G* space. Then, IF estimation method by applying first and second derivative tests identifies the local extremums (with respect to frequency). In this step, valid peak are the local extermums which have values higher or lower than a predefined threshold. Finally, for detecting the linked components an algorithm specifically designed for detection of linking component is applied by evaluating the connectivity of the pixels and also the number of the connected pixels. The fact behind this is that IF of a component of a signal (where energy of the signal is concentrated) is observable in the TFD space as a ridge which describes the IF.

Selection of a proper TFD representation approach for representing gait cycle can be counted as the first step in designing any identification system using TF space. A proper TFD method is the one which is capable of emphasizing the non-stationarities of the given signal which, in turn, gives the system highest discriminative power to correctly discriminate between different cases in the population under consideration. In this study we use smoothed Wigner-Ville distribution (SWVD) as it is capable of reducing the cross-term affection while it provides good resolution [31,32]. SWVD is a variant method which incorporates smoothing by independent windows in time and frequency, namely (τ) and (t):

(5) $$SPWV\left(t,\omega \right)=\int_{-\infty }^{+\infty }W_{\omega }\left(\tau \right)\left[\int_{-\infty }^{+\infty }W_{t}\left(u-t\right)x(u+\frac{\tau }{2})x^{*}\left(u-\frac{\tau }{2}\right)du\right]e^{j\omega \tau }d\tau$$

The feature extraction using SWVD is based on Energy, Frequency and Length of the principal track. Each segment gives the values $E_{k}$ (energy), $F_{k}$ (frequency), and $L_{k}$ (length). The signal is firstly divided into segments; then, the construction of a three-dimensional feature vector for each segment will take place. Energy of each segment can be calculated as follows:

(6) $$E_{k}=\int \int_{-\infty }^{+\infty }\vartheta_{k}\left(t,f\right)dtdf$$

where $\vartheta_{k}\left(t,f\right)$ stands for the time-frequency representation of the segment. However, to calculate the frequency of each segment *k*, we made use of the marginal frequency as follows:

(7) $$F_{k}=\int_{-\infty }^{+\infty }\vartheta_{k}\left(t,f\right)dt$$

**Figure 5:**
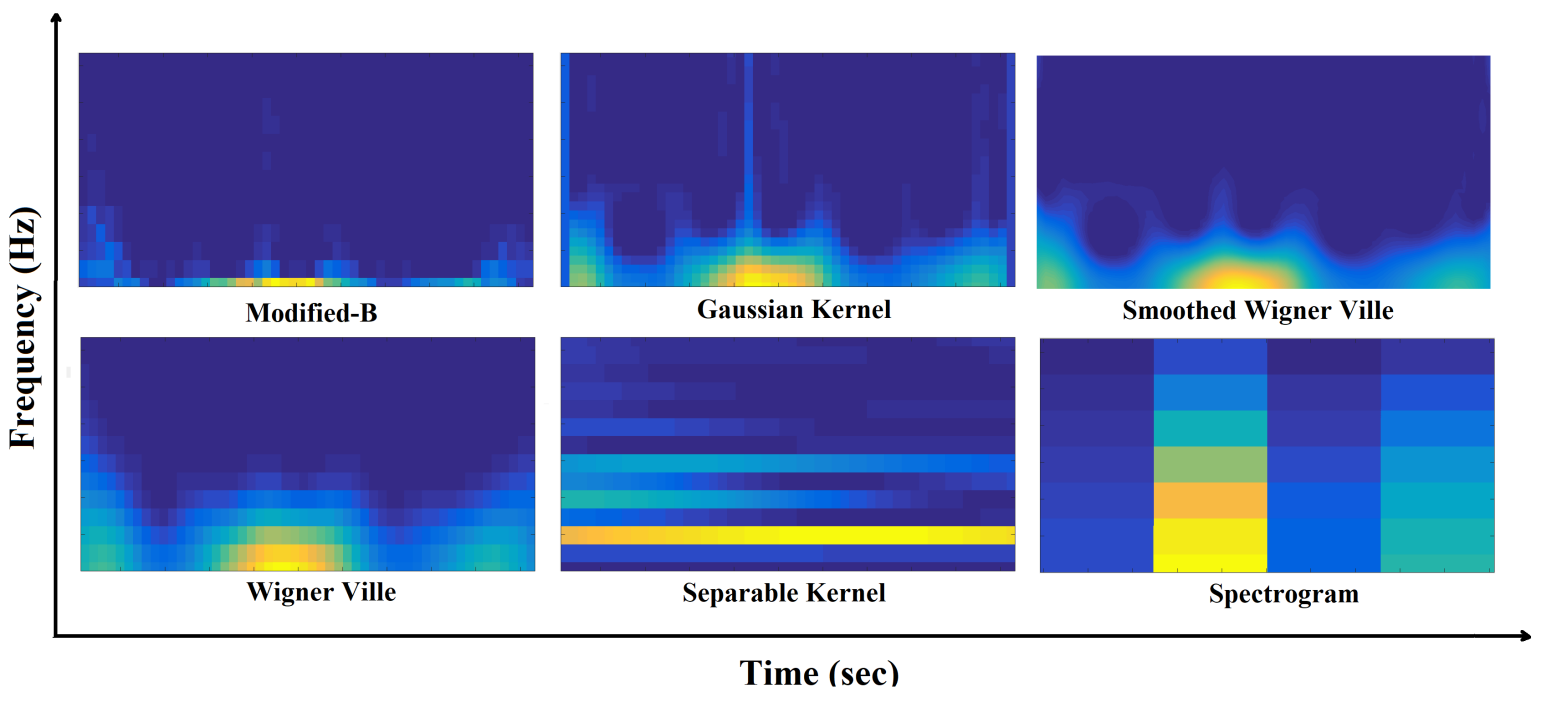
Time-frequency representation of one cycle data using different TFDs.

For the purpose of SWVD representation we use a MATLAB toolbox designed by François Auger at CNRS (France) and Rice University (USA) [32]. Figure 5 illustrates the TF representation using 6 different TF approaches.

### 2.5. Deep Convolutional Neural Networks (DCNN)

**Figure 6:**
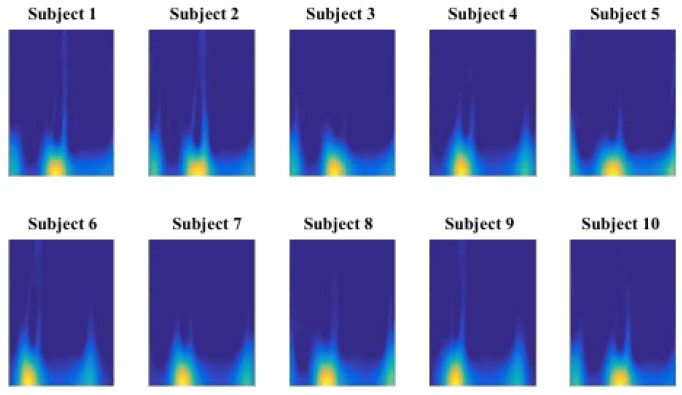
TF representation of gait samples as CNN inputs.

Figure 6 demonstrates an example TF representation of gait cycle for 10 different subjects. Taking a close look at the gait cycle data, it is evident that the temporal orientation and order of the data stand out as significant discriminative features. We design a Convolutional Neural network (CNN) structure as a deep hierarchical structure for feature extraction and predictive modeling. We intend to verify if multiple feature maps generated by CNN process would preserve the temporal aspect of the gait cycle data and provide higher level of discriminative feature space.

CNNs are most commonly used in pattern recognition. A simple CNN is a sequence of steps where each step transforms a volume of activations through a set of functions that are differentiable. They are made up of neurons with learnable weights and biases [33]. Though the weight vector optimization is similar to the conventional neural networks. CNNs are designed to deal specifically with 2D or 3D image data. A variety of combinations of linear and non-linear differentiable steps could be used to build a deep CNN and that determines the complexity of the system [34].

We have developed a non-parametric fully supervised DCNN model for motion-based gait authentication. The model takes a 3D input image $x_{i}$ and transforms it into a prediction probability vector $\bar{y_{i}}$ for ten different classes which correspond to the 10 participating subjects. We train the model using *N* labeled images {x,y} where the label $y_{i}$ is the class label of the input data. Training minimizes a SoftMax loss function with respect to network parameters such as weights and biases using a gradient descent method and network parameters are updated using back propagation.

We aim to conduct a comparison between the sensor locations in terms of how well each can describe subject dependent gait signatures towards the identification task. Furthermore, we aim to investigate multi-sensor fusion in order to enhance the gait identification performance using complementary information among various sensors.

#### DCNN Architecture

We have used the following four main building blocks in the DCNN model:

- Convolution.
- Pooling.
- Rectified linear unit (ReLU).
- Fully connected layer.

The convolution layer performs convolution of input with a set of predefined filters, which is a dot product between the filters and the region they are connected to in the input image. If we consider *k* kernels of spatial dimensions’ $h_{k}$ and $w_{k},$ then the filter tensor would have the dimension of $h_{k}$ × $w_{k}$ × *k* × $k^{\prime }$ which could be represented by tensor ***w***. Considering a simple convolution layer with zero padding and a unit stride, the output *y* after performing convolution on an input layer of tensor *x* could be represented as

(8) $$y_{i^{\prime }j^{\prime }k}=\sum_{i=0}^{h_{k}}\sum_{j=0}^{w_{k}}\sum_{k^{\prime }=0}^{k}w_{ijk^{\prime }k}\times x_{i+i^{\prime },j+j^{\prime },k^{\prime }}$$

In Equation (8), $x_{i+i^{\prime },j+j^{\prime },k^{\prime }}$ represents the $\left(i+i^{\prime },j+j^{\prime },k^{\prime }\right)$ indexed element of the input tensor *x*.

The pooling layer applies a chosen operator and combines closely associated feature values. It is used to down sample the input image along the width and height. This layer does not require parameter learning. A simple implementation of max pooling can be represented as

(9) $$y_{i^{\prime }j^{\prime }k}=\;{}_{0\;\leq i\;\leq h_{p},\;\;0\;\leq j\;\leq w_{p}}^{Max}\;\;\{x_{i^{\prime }*h_{p}+i,\;\;j^{\prime }*w_{p}+j,\;\;k}\}$$

where *x* and *y* represent the $i^{\prime }j^{\prime }k$ indexed input and output layer and $h_{p}$, $w_{p}$ are the pooling window dimensions.

The Rectified Linear Unit (ReLU) is a non-linear activation layer introduces the non-linearity when applied to the feature map. ReLU layer leaves the size of its input unchanged. A simple implementation of ReLU would be as below:

(10) $$y_{i^{\prime }j^{\prime }k}=\max\left\{0,x_{i^{\prime }j^{\prime }k^{\prime }}\right\}.$$

where *x* and *y* are input and output of corresponding tensors. Like pooling layer, ReLU does not need any parameter learning and it does not alter the dimensions of the input layer.

## 3. Multi-Sensor Fusion

**Figure 7:**
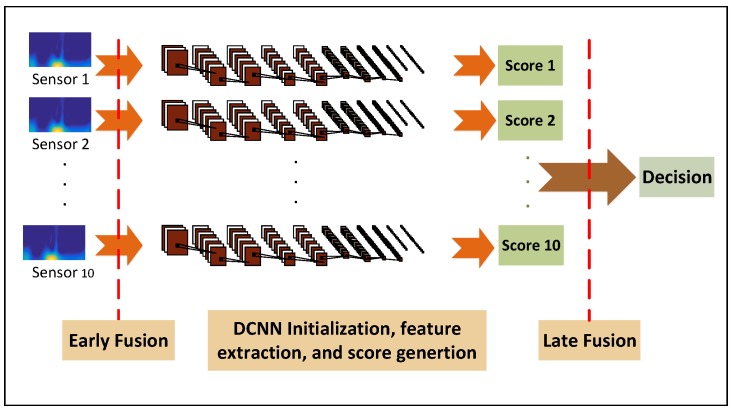
block diagram for early and late score fusion.

In this work, we investigate 5 different IMU sensor locations on the body and evaluate them for the gait identification task. Besides the comparison between the sensor locations in terms of how well each can distinguish subjects based on their gait, we also aim to investigate multi-sensor fusion in order to incorporate complementary information among various sensors to enhance the gait identification performance. The sensor fusion system is generally grouped into two types, namely early and late fusion. The basic idea of fusion is to combine multiple decisions generated by different experts in an attempt to enhance the performance of the overall system. A key issue to design a suitable and effective fusion scheme is to appropriately exploit all the available discriminative cues to generate an enhanced identification performance. Information fusion can be carried out at three levels of abstraction closely connected with the flow of the classification process: (1) data level fusion; (2) feature level fusion; and (3) model score fusion [35]. In this work, we present a simple data level fusion by concatenating the TFs from different sensor nodes and modalities into one collective input to the DCNN structure. We then propose and investigate an iterative minimum classification error multi-score fusion algorithm. Figure 7 demonstrates a block diagram of the proposed early and late fusion.

### 3.1. Early Fusion

Sensor fusion at the input level was first considered in this study. We aim to investigate multi-sensor fusion in order to enhance the gait identification performance using complementary information among various sensors. The key advantage of CNN is the feature learning ability, which can automatically discover an intricate structure and learn useful features from raw data layer by layer, which enables CNN to fuse input raw data and extract basic information from it in its earlier layers, fuse the basic information into higher level representation of information, and further fuse those information in its later higher layers to form the final classification result. For early fusion, we aggregate the information in all the synchronous IMUs at the input level to feed the DCNN model. The input level fusion is achieved by combining the 3D gait cycle images from five different sensors to form a 120×120×30-dimensional image. The intuition behind early fusion is to incorporate all possible information that various sensors generate in order for the DCNN learning structure to learn the discriminative features in an iterative manner.

### 3.2. Late Fusion

The early-fusion method fuses two or more sensor readings by combining their transformed 2D gait cycle data. It provides a comprehensive input space to discover higher performance predictors. However, the input space grows in dimensions via early fusion and therefore, the search space for the best predictor increases exponentially. On the other hand, DCNN is a gradient descent learning method for the best predictor, which has serious limitations when the search space grows. Also, early fusion often cannot handle incomplete measurements. If one sensor modality becomes useless due to malfunctions, breakdown or etc, its measurements will be rendered ambiguous. In this section, we investigate late fusion of multiple sensor nodes (i.e., five inertial sensors) and modalities (i.e., Accelerometer and Gyroscope readings). In this way, a DCNN is independently trained to perform human gait identification based on each sensor location and modality. Subsequently, the one-vs.-rest discriminative output scores are fused at to generate the combined gait identification decision. We aim to design discriminative fusion of classification scores generated by individual DCNNs to reduce the classification error rate in the gait identification task. The fusion of multiple model scores operates as a mixture of experts that makes a collective decision by exploiting complementary information from each of the individual identification models. The input to each different classifier is generated by the TF spectro-temporal front-ends. If the model outputs associated with the different IMUs offer complementary information, multi-sensor fusion can improve the performance.

In this way, we generate the posterior probability scores from 10 DCNNs trained on the resulting factors of accelerometer and Gyroscope readings from five sensor nodes, individually. We introduce a discriminative performance measure to optimize the performance of the fusion of the 10 DCNNs. We propose the minimum error score fusion (MESF) method that iteratively estimates the parameters of a linear fusion with the aim to minimize the identification error rate on the training data. The proposed iterative algorithm learns the score weights one by one by finding the optimum decision threshold of each DCNN-i, {i|i=1,…,N} for each specific target subject.

### 3.3. The Minimum Error Score Fusion (MESF)

The human gait identification task is to recognize *M* target subjects using *N* individual gait classifiers (i.e., DCNNs). The *i*-th DCNN maps the gait segment **x** to a score vector $S_{i}\left(x\right)=\left\{S_{i,j}\left(x\right)|j=1,2,ߪ,M\right\}$, in which each element, $S_{i,j}\left(x\right)$, is the log-likelihood score of **x** associated with the DCNN *i* and target subject *j*. The fusion output score for the target subject *j*, $Score_{j}\left(x\right)$, is calculated by combining the output scores of the DCNN sub-systems,

(11) $$Score_{j}\left(x\right)=\left\{\sum_{i=1}^{N}w_{i,j}S_{i,j}\left(x\right)\right\},j=1,2,\ldots ,M$$

where $w_{i,j}$ is the weight corresponding to $S_{i,j}\left(x\right)$. However, unlike conventional linear score weighting techniques, the weighting coefficients are subject-dependent in which, the weighting coefficients vary for different subjects and individual DCNNs. By doing so, it is expected that the inter-subject discriminative information is taken into account. It also may reflect how one DCNN sub-system contributes to identify each particular subject. There are a total of N×M weighting coefficients as fusion parameters to be estimated using the MESF algorithm. We then apply a normalization process on the scores of *M* target subjects.

#### 3.3.1. The Process of Score Fusion

**Figure 8:**
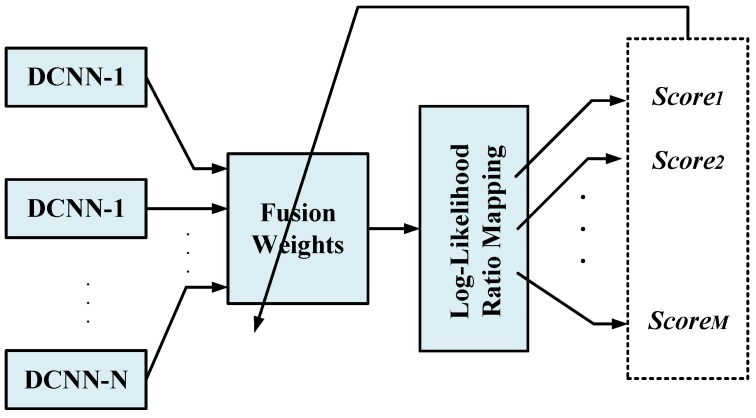
The training paradigm for learning the fusion weights.

Figure 8 shows the process of learning the fusion weights. The MESF learning algorithm uses error feedback from the final fusion decision to fine-tune the fusion weights. We propose and employ a discriminative measure to find the optimum decision threshold in a one-vs-rest manner to learn the fusion weights of the general *M*-class identification problem. The gait recognition results are reported as the weighted average over multiple subject identifier DCNNs.

As the distribution of the decision scores for each DCNN might be different due to different node locations and modalities, the scores are less compatible across different DCNN models. Hence, we conduct score normalization to provide consistency over the output scores of the DCNN models. The $Score_{\varphi }\left(x\right)$ from Equation (11) is converted to log-likelihood ratio (LLR) $\hat{S}core_{\varphi }\left(x\right)$ as presented in [36].

(12) $$\hat{S}core_{\varphi }\left(x\right)=Score_{\varphi }\left(x\right)-log\langle \frac{1}{M-1}\sum_{j=1,j\neq \varphi }^{M}exp\left(Score_{j}\left(x\right)\right)\rangle$$

The input segment **x** is classified as the target subject φ if,

(13) $$\hat{S}core_{\varphi }\left(x\right)>\theta_{\varphi }$$

where $\theta_{\varphi }$ is the decision threshold for the target subject φ, which is to be learned using cross validation on the training data. The weight $w_{i,\varphi }$, corresponding to the DCNNs output score $S_{i,\varphi }\left(x\right)$, can be interpreted as the degree to which $S_{i,\varphi }\left(x\right)$ contributes in the identification decision. From Equation (13), the fusion weight $w_{i,\varphi }$ can be directly related to the final decision as follows,

(14) $$w_{i,\varphi }>\frac{\theta_{\varphi }+log\langle \frac{1}{M-1}\sum_{j=1,j\neq \varphi }^{M}exp\left(-Score_{j}\left(x\right)\right)\rangle -\sum_{k=1,k\neq i}^{N}w_{k,\varphi }S_{k,\varphi }}{S_{i,\varphi }\left(x\right)}$$

That is, $w_{i,\varphi }$ needs to satisfy Equation (14) in order for the input segment **x** to be identified as the target subject φ. The MESF directly relates the performance of the system to the fusion parameters and determines the weights one at a time iteratively by solving a one-vs-rest subject identification problem to minimize the errors of the model score fusion.

#### 3.3.2. The MESF Algorithm for Fusion Weight Learning

(1) Discriminative performance measure of the linear fusion:

DCNN models usually assign a score $S\left(x\right)$ to each unseen gait segment x, expressing the degree to which x would belong to each of the subject classes. Ideally, the score is an accurate estimate of posterior probability distributions over the target subject class (i.e., positive class), $p{(}^{\prime }pos^{\prime }|x)$, and the rest of the subjects (i.e., negative class), $p{(}^{\prime }neg^{\prime }|x)$, for an input **x** which denotes the estimated probabilities that **x** belongs to the positive and negative classes, respectively. We introduce a measure, Θ(x) to convert the probabilities into discriminative scores as below:

(15) $$\Theta \left(x\right)=\frac{p{(}^{\prime }neg^{\prime }|x)}{p{(}^{\prime }pos^{\prime }|x)}$$

The measure Θ(x) demonstrates the the degree to which **x** is predicted to be of the negative class). By optimizing a threshold on the Θ measure, a statistical classifier can be converted to a discriminative classifier. In this way, the input **x** is classified as negative if the Θ(x) is greater than a specified threshold and positive otherwise. The desired threshold is learned by minimizing the classification error rate on the training data.

(2) Minimum error rate decision threshold in one-vs.-rest manner:

Assume that a training set $\left\{\left(x_{t},y_{t}\right)|t=1,2,\ldots ,n\right\}$ consisting of *n* labeled data and their Θ measure $\Theta (x_{t})$ is available. Using the training data, we aim to design an algorithm that finds the value of the linear fusion weight parameter that makes the optimum decision on a set of labeled training data samples. The proposed algorithm aims to return the decision threshold ‘optimum_thresh’ that minimizes the error rate of the linear classifier fusion by varying the threshold value from 0 to +∞ and find the optimum value by including false negatives (FN) in the positive class. Therefore, we rank the data samples in an ascending order of their Θ(.) measure $\Theta \left(x_{1}\right),\ldots ,\Theta \left(x_{P+N}\right)$. Considering any threshold, th, between $\Theta (x_{t})$ and $\Theta (x_{t+1})$, the first *K* data samples will be classified as positive, where $\Theta (x_{K})<th$, and the remaining *P + N − K* data samples as negative. In this way, a maximum of *P* + *N* + 1 different thresholds has to be examined to find the optimum decision threshold. The first threshold classifies everything as negative and the rest of the thresholds are chosen in the middle of two successive Θ measures.

The threshold, optimum_thresh, on the Θ(.) measure is found such that it minimizes the error rate of the DCNN model score fusion.The data sample $x_{t}$ is classified as positive if $\Theta (x_{t})\leq optimum\_thresh$. That is, $x_{t}$ is classified as ${}^{\prime }pos^{\prime }$ if,

(16) $$\frac{p(x{|}^{\prime }neg^{\prime })}{p(x{|}^{\prime }pos^{\prime })}<optimum\_thresh\;\;\;\;\to \;\;\;\;p\left(x{|}^{\prime }neg^{\prime }\right)<optimum\_thresh\times p\left(x{|}^{\prime }pos^{\prime }\right)$$

The algorithm results in the highest accuracy achievable with the given Θ(.) on the given set of data samples. In practice, the quality of the optimization depends on the quality of the positive and negative scores, e.g., the output posterior probability distributions of the DCNN models. In our fusion task, if the individual DCNN models generate good quality estimates, the fusion weight learning can considerably improve the performance. It can also be implemented in an efficient way by exploiting the monotonically ranked Θ measures.

(3) Discriminative learning of the fusion score weights

We propose an iterative learning procedure in order to minimize the error of the overall human identifier in one-vs-rest manner by re-estimating the weight parameters of the model score fusion. The proposed learning method adjusts the fusion weights in the interval [0,∞) using the training gait segments. The weights assigned to the output scores $S_{i,j}\left(.\right)$ are set to one, for $w_{i,j}\leftarrow 1$ for i=1,…,N, and j=1,…,M, as an initial solution to the problem. Then, the following procedure is presented, in which the error rate of the DCNN model fusion is successively reduced by finding a better solution than the current one by learning the locally optimum fusion weights, one at a time. To find the weight $w_{i,\varphi }$, corresponding to the output score of the DCNN *i* for target subject φ, the problem is considered to be a 2-class problem where the target subject φ is the positive class and $\bar{\varphi }$ comprising the rest of the subjects is the negative class. The steps are as follows:

1. The $w_{i,\varphi }$ is set to zero, i.e., $S_{i,\varphi }\left(.\right)$ does not contribute in classification decision.
2. The gait segments in the training set belonging to the subject φ that are classified correctly with current values of the model score weights are marked. Adjusting the weight $w_{i,\varphi }$ does not have any impact on their classification.
3. The gait segments of $\bar{\varphi }$ in the training set that are misclassified are marked. Adjusting the weight $w_{i,\varphi }$ does not have any impact on their classification.
4. The remaining *m* gait segments in the training set $\left\{x_{t}|t=1,\ldots ,m\right\}$ that are left unmarked are used to determine $w_{i,\varphi }$ so that the error of the model score fusion is minimized over $\left\{x_{t}|t=1,\ldots ,m\right\}$. From Equation (14), the measure $\Theta_{i,\varphi }\left(.\right)$ is calculated for every segment in $\left\{x_{t}|t=1,\ldots ,m\right\}$ as follows:
  (17) $$\Theta_{i,\varphi }\left(x_{t}\right)=\left[\theta_{\varphi }+log\langle \frac{1}{M-1}\sum_{j=1,j\neq \varphi }^{M}exp\left(-Score_{j}\left(x_{t}\right)\right)\rangle -\sum_{k=1,k\neq i}^{N}w_{k,\varphi }S_{k,\varphi }\left(x_{t}\right)\right]/S_{i,\varphi }\left(x_{t}\right)$$
  where, $\Theta_{i,\varphi }\left(x_{t}\right)$ is the amount of $w_{i,\varphi }$ necessary for $x_{t}$ to be classified as the target subject φ.
5. The training segments are ranked in an ascending order by their $\Theta_{i,\varphi }\left(.\right)$ measure. A threshold is defined and initialized to zero. Then, assuming that $x_{t}$ and $x_{t+1}$ are two successive segments in the list, the threshold is computed as,
  (18) $$thresh=[\Theta_{i,\varphi }\left(x_{t}\right)+\Theta_{i,\varphi }\left(x_{t+1}\right)]/2$$
  Then, the threshold is adjusted from the lowest score to the highest. For each threshold, the associated accuracy of the score fusion is measured. The value of the optimum_thresh leading to the maximum accuracy is used as the estimated score weight $w_{i,\varphi }$, assuming that all other score weights are fixed.

**Figure 9:**
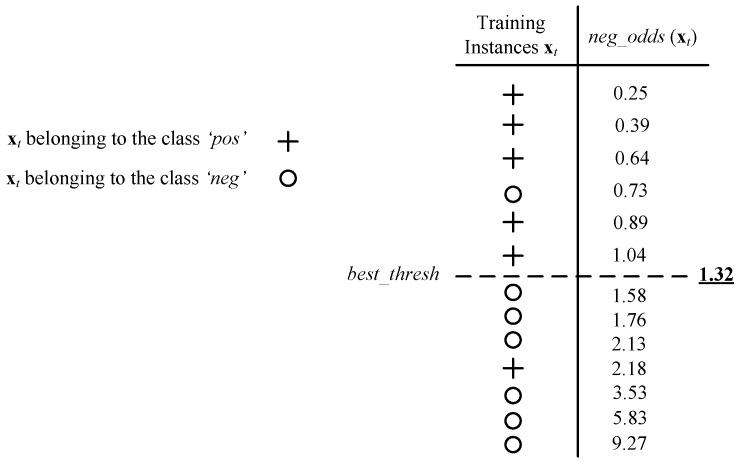
A data sample of the resulting thresholds on the $\Theta_{q,w}\left(.\right)$ measure.

Figure 9 shows a data sample of a resulting threshold on the $\Theta_{q,w}\left(.\right)$ measure leading to the highest accuracy. In this way, the input $x_{t}$ is classified as *w* if its $\Theta (x_{t})$ is lower than the threshold. The algorithm determines the optimal value of the parameter $\phi_{q,w}$ assuming that all other parameters are given and fixed.

The search for the locally optimum combination of weights is conducted by optimizing the score weights one at a time and learning stops if no improvement to the current performance can be made. Based on our observations, the above method consistently generated superior results. The MESF method determines the decision score weights of the sub-systems in order to better discriminate between the gait segments from subjects φ and those of the rest by finding the optimum decision threshold of the score fusion in the one-vs-rest manner. By doing so, we can interpret that the ROC curve of the score fusion is locally optimized. As shown below, it is illustrated that the error rate on the training data always reduces during the optimization and will converge to a minimum point.

(19) $$Error_{Rate}\left(\Lambda \cup \Phi_{q,w}^{new},D\right)\leq Error_{Rate}\left(\Lambda \cup \Phi_{q,w}^{old},D\right)$$

where Λ is the set of all other parameters (Given & Fixed)

(20) $$\Rightarrow Error_{Rate}\left(\Lambda \cup \Phi_{q,w}^{t},D\right)\leq Error_{Rate}\left(\Lambda \cup \Phi_{q-1,w}^{t-1},D\right)\geq Error_{Rate}\left(\Lambda \cup \Phi_{q-2,w}^{t-2},D\right)\leq \ldots$$

where *t* represent the iteration number starting from 1 and increment after each learning iteration. After each iteration, the accuracy of the learning algorithm decreases.

### 3.4. Comparable Score Fusion Methods

In this section, some other linear score fusion methods are described for comparison. Assume an input gait segment **x**, and the output decision scores of the sub-systems, $\left\{S_{i,j}\left(x\right)|i=1,\ldots ,N\;\;\&\;\;j=1,\ldots ,M\right\}$ are available. Comparative methods include a simple non-trainable combiner and the large margin method that require a more sophisticated training procedures.

- Summation: The output scores of the individual DCNNs are summed up and the subject that receives the highest score is the output decision of the fusion system.
  (21) $$Score_{j}^{\mathrm{sum}}\left(x\right)=\sum_{i=1}^{N}S_{i,j}\left(x\right)$$
- Support vector machine (SVM): SVM has shown to be effective in separating input vectors in 2-class problems [37], in which SVM effectively projects the vector **x** into a scalar value f(x),
  (22) $$f\left(x\right)=\sum_{i=1}^{N}\alpha_{i}y_{i}\left(x_{i}.x\right)+d$$
  where the vectors $x_{i}$ are support vectors, $y_{i}=\left\{-1,1\right\}$ are the correct outputs, *N* is the number of support vectors, (.) is the dot product function, $\alpha_{i}$ are adjustable weights, and *d* is a bias. Learning is posed as an optimization problem with the goal of maximizing the margin, which is the distance between the separating hyperplane and the nearest training vectors. As a combiner method, the output scores of all the DCNNs are concatenated to form a vector as the input to SVMs. Then, one SVM is assigned to each of the *M* target subjects, the one-vs-rest scheme, and train accordingly to get the final output scores,
  (23) $$Score_{j}^{\mathrm{SVM}}\left(x\right)=\sum_{i=1}^{N}\alpha_{i}y_{i}\left(\phi \left(x_{i}\right).\phi \left(x\right)\right)+d$$
  where ϕ(x) is the vector resulted by concatenating the output scores of all the DCNNs for the input segment **x**.

## 4. Results

We implemented a Matlab TF representation toolbox [38] to generate the SWVD TF representation of the data and use Matlab print function to output a 2D image with specific resolution and size. After experimenting with multiple image sizes, it was observed that higher image resolution did not necessarily mean better model performance. DCNN model was tested with different input image sizes. The image size was modified by changing the resolution of the figure generated by Matlab print function. It was observed that the model prediction accuracies are highest with the image size of 120×120×3 for our current analysis.

**Figure 10:**
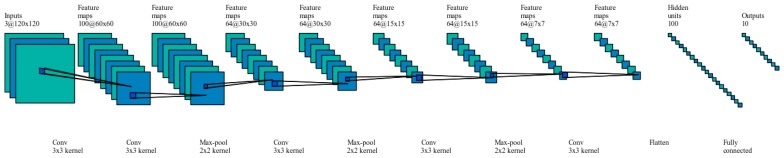
CNN architecture.

For DCNN model designed in this study, the architecture guidelines as mentioned in [39,40] were followed. Small filters were used and the image size was reduced using higher stride lengths where necessary. Padding was introduced in the convolution layers to prevent convolution layer from altering the spatial dimensions of the input. The spatial dimensions were altered in pooling layer by down sampling. Also, more ReLU activation layers were used across the DCNN after each convolution and pooling pair to bring in element wise non-linearity. The current model consists of 10 (CONV, RELU, POOL) layers and one fully connected layer as shown in Figure 10.

### 4.1. Model Selection and Evaluation Procedure

We consider the human gait identification task as a pattern recognition problem, in which a set of training samples was employed for gait signature pattern extraction and predicitve model training, a separate set of validation samples was used for hyperparameter tuning, and an exclusive set of test samples was used for predictive model evaluation. In order to estimate the generalization accuracy of the predictive models on the unseen data, 10 fold cross validation (10-CV) was used. 10-CV divides the total input data of *n* samples into ten equal parts. In every iteration one part is considered to be a test sample set and the remaining nine parts are considered to be a validation and training sample set. There is no overlap between the test sample set (10% of data) with the validation and training sample set (90% of data). The latter set is further divided into 4:1 ratio of training and validation data samples. The sets were permuted over 10 iterations to generate an overall estimate of the generalization accuracy. For an input data set of *n* samples:

- Number of Test Samples: *n*/10
- Number of Validation samples: ($n-\frac{n}{10}$) × 1/5
- Number of Training samples: ($n-\frac{n}{10}$) × 4/5.

Data set has *n* = 4178 samples, which includes images from ten different subjects. Each iteration of the 10—fold cross validation would have the following division of data (Note that there was no overlap between the training set, the validation set, and the test set in all the iterations of 10-CV).

- Number of Test Samples: 417
- Number of Validation samples: 752
- Number of Training samples: 3008

Figure 6, shows a sample image set of all subjects for sensor#1. The DCNN model was trained using the training and validation set and tested independently with the testing set. In order to fine-tune the parameters of the DCNN model, we conducted random restart hill climbing search method on a low resolution quantized parameter space to achieve a reliable local minimum in the error function (i.e., the average error rate of the model on the 5 individual sensor locations). Table 2 reports the selected parameters to train the gait identification DCNN models. It must be noted that no of epochs was set to 19 since, we terminate training at 19 epochs where we get maximum accuracy and the model is able to generalize enough and avoids overfitting.

### 4.2. Individual Sensor Performance

**Figure 11:**
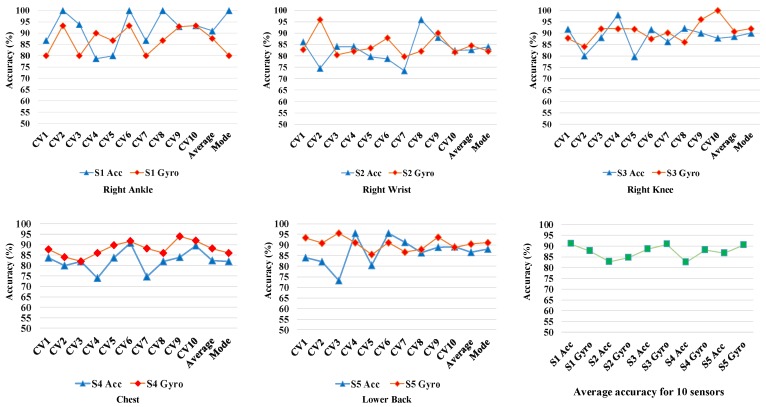
10 Cross Validation Performance of the Individual Sensors Including Both Gyroscope (Gyro) and Accelerometer (Acc) sensors. $CV_{j}$ indicates Cross-Validation *j*, and $S_{i}$ indicates sensor *i*.

The prediction accuracies of the DCNN models for each iteration of the 10-CV and for different sensors are shown in Figure 11. It demonstrates the average accuracies achieved using each of the five individual sensors over the 10 iterations of 10-CV. The blue and red markers represent the accuracies corresponding to the accelerometer and gyroscope data recorded from each sensors, respectively. It can be observed that the gyroscope generated data (i.e., angular velocity) of the sensors are better predictors than the acceleration data in 3 out of 5 cases. Only the acceleration data from sensor#1 (on the right ankle) and sensor#2 (on the right wrist) exhibited higher gait identification accuracy compared to angular velocities from the same sensors. It might be due to wider range movements of the ankles and wrists generating more individually descriptive accelerations. The acceleration data from sensor#1 on the ankle generates the best average 10-CV accuracies among all 10 different sources of data (Acc No.). This observation was expected since dynamics of gait is mostly captured by the lower limb movement [41]. For the gyroscope data, the average 10-CV prediction accuracy for different sensors in decreasing order is as follows: sensor#5 (lower back), sensor#3 (right knee), sensor#4 (chest), sensor#1 (right ankle), sensor#2 (right wrist). For the accelerometer data, prediction accuracies of the sensors in descending order is: sensor#1, sensor#3, sensor#5, sensor#2, sensor#4. The ranking of the sensors in terms of identification accuracies are not consistent for gyroscope and accelerometer sensors. It loosely suggests that upper trunk locations is more suitable for Gyroscopes and lower limb locations are more suitable for accelerometers. The accelerometer at the at the right ankle sensor (capturing high range lower limb acceleration) and the gyroscope sensor of of the lower back sensor (capturing the angular velocity of trunk movements) generated the highest accuracies. This observation suggests that incorporating the complementary discriminative information generated from different sensors and modalities can be fused and improved. Therefore, multi-sensor fusion is investigated in early and late fashions in order to improve the the recognition performance.

### 4.3. Early Fusion

**Figure 12:**
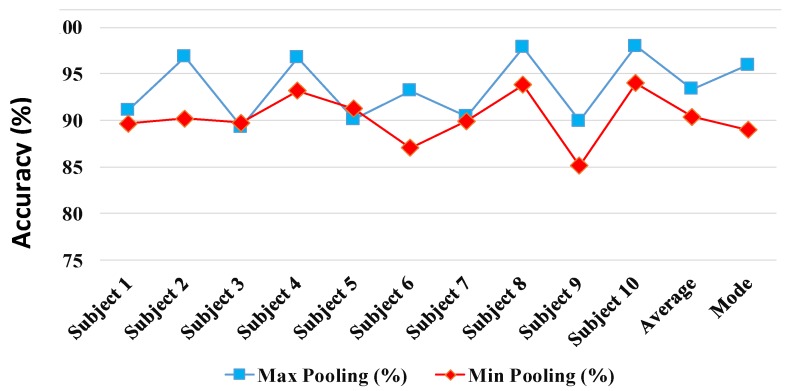
Multi-sensor early fusion performance using min and max pooling.

In early fusion, we investigate aggregating the complementary discriminative motion data recorded synchronously from the five IMUs. In this way, we conduct a sensor fusion at the input level to the DCNN model as discussed in Section 3.1. The input level fusion is achieved by combining the 3D gait cycle images from five different sensors to form a 120×120×30–dimensional image. The average subject identification accuracy percentages of the DCNN model, whose parameters are optimized using 10-CV cross validation method, is shown in the Figure 12. The early fusion via input image aggregation at the input to one overall DCNN demonstrated improved prediction accuracies as shown in Figure 12. Results report that using Max pooling is preferable over Min pooling leading to higher identification accuracy in 8 out of 10 subject cases. The average 10-CV gait identification performance achieved, using Max pooling, was 93.36%. The results suggests that using a simple input aggregation fusion of sensors by aggregation can enhance the performance of the best DCNN model (i.e., Sensor#1 Acc with 91.01% accuracy) with 26.2% relative improvement. In order to compare the results of the early fusion model performance and Sensor#1 Gyro DCNN model, we conducted 10-CV test 10 times and run a statistical test with the null hypothesis that the performance accuracies of the fusion and ’S1 Acc’ on different subjects comes from independent random samples from normal distributions with equal means, using the two-sample t-test. The test rejected the null hypothesis with the *p*-value = 0.028 which demonstrate significant improvement over the best individual sensor-based DCNN model under the 95% confidence interval.

### 4.4. Late Fusion

For late fusion, we employ the MESF algorithm described in Section 3.3. The discriminative measure to find the optimum decision threshold in one-vs-rest manner introduced in Section 3.2 is utilized to learn the fusion weights of the general *M*-class identification problem (M=N=10). Therefore, the total number of weight coefficients as fusion parameters to be estimated are N×M=50. We applied log likelihood score normalization in order to provide consistency over the output scores of the individual DCNNs. Table 3 presents the results for score level fusion using the MESF algorithm. Table 3 reports that MESF using Max pooling lead to higher identification accuracy compared to Min pooling in 9 out of 10 subject cases. As Table 3 reports, the proposed MESF method generates the average 10-CV accuracies of 97.06% and 95.24% using Max pooling and Min pooling, which lead to 67.3% and 47.1% relative improvement compared to the best individual DCNN results, respectively.

Using the MESF fusion algorithm, we achieved the highest 10-CV accuracy of 97.06% on the 10-class identification problem with 10% expected accuracy of random guess. In the case of having a larger subject population, its impact will be solely on the number of impostors per each one-vs-rest subject identifier (the number of targets will remain the same). That will limit the potentially introduced errors to false alarms. On the other hand, our proposed method in this paper is designed to minimize the one-vs-rest identification errors. By decoding the gait cycles via spectro-temporal expansion, we discover and capture discriminative individual low and high level signatures and predictive model jointly via the trained DCNN structure and then discriminatively multi-sensor fusion model. Our aim with the error minimizing design of joint our proposed models was to equip them to cope with both false alarms and miss detections.

### 4.5. Comparative Study

We also compare the proposed MESF method with two other linear score fusion methods, namely SUM and SVM methods introduced in Section 3.4. Table 3 presents the results for our MESF method, SVM, and SUM using Max and Min pooling. According to Table 3, Max pooling performs better in majority of cases for various subjects and fusion methods. SUM method did not demonstrate improvements in either cases of Max and Min pooling compared to the best individual DCNN. It might be due to the fact that the lower accuracy DCNN model scores adversely affect the fusion identification decisions. On the other hand, SVM fusion method generates improved results in case of Max pooling via solving an optimization problem with the goal of maximizing the margin between the target subjects and the others. In this way, one SVM is assigned to each of the 10 target subjects (i.e., one-vs.-rest scheme). We applied the two-sample t-test to evaluate the significance of improvement of SVM fusion compared to the best individual DCNN. The test rejected the null hypothesis with the *p*-value = 0.034, which is significant under 95% confidence level. Although the performances of the SVM fusion relatively improves the best individual performance by 25.2%, they are inferior to the MESF performance.

## 5. Conclusions

In the human gait recognition task, the aim is to extract discriminative features and descriptors from gait motion signals to identify our target subject from others. The manual feature extraction is prone to error due to the complexity of data collected from inertial sensors and the disconnection between feature extraction and the discriminative learning models. To overcome this shortcoming, we proposed a novel methodology for processing non-stationary signals for the purpose of human gait identification. The proposed methodology comprises four main components: (1) cycle extraction; (2) spectro-temporal 2D expansion and representation; (3) deep convolutional learning; and (4) discriminative multi-sensor model score fusion. We first isolated the gait cycles using a simple and effective heuristic. Then, we conducted spectro-temporal transformation of isolated gait cycles. The 2-D expansion of the gait cycle increased the resolution of the desired discriminative trends in the joint time-frequency domain. In order to avoid manual feature extraction and incorporate joint feature and model learning from the generated high resolution 2D data, we designed a deep convolutional neural network structure in order to process the signal layer by layer, extract discriminative features, and jointly optimize the features and the predictive model via error back propagation model training. We investigated 5 IMU sensor placement on the body and conducted a comparative investigation between them in terms of gait identification performance using synchronized gait data recordings from 10 subjects. Due to complementary discriminative signature patterns captured from the recorded signals collected via different sensors, we then investigated gait identification fusion modeling from multi-node inertial sensor data and effectively expand it via 2-D time-frequency transformation. We perform early (input level) and late (model score level) multi-sensor fusion to improve the cross validation accuracy of the gait identification task. We particularly proposed the minimum error DCNN model score fusion algorithm. Based on our experimental results, 93.88% and 97.06% subject identification accuracy was achieved via early and late fusion of the multi-node sensor readings, respectively.

## 6. Future Research Direction

In this paper, the problem of human gait identification was investigated under the bigger umbrella of ubiquitous and continuous IMU-based gait analysis work group at the Wearable Sensing and Signal Processing (WSSP) lab. Our main contribution and focus in this paper was our proposed model score fusion algorithm to incorporate the complementary discriminative scores generated by each individual DCNN model given a input human gait cycles. We used a local search and an overall accuracy to generate a reliable baseline DCNN model for all the IMU recordings. Due to the fact that data from different sensors (i.e., sources) may have different characteristics, the DCNN training paradigm can be improved by sensor-dependent tuning for different sensor locations and modalities. Therefore, our team is currently investigating sensor location and modality specific DCNN model optimization and subsequently, designing multi-sensor and multi-model fusion algorithms. We are also increasing the number of subjects and the walking conditions in our data set. When completed, our plan is to create a link on our website to share our data set and our baseline model implementations with the research community.

## Figures and Tables

**Table 1:** The parameter values of the Gyroscope and Accelerometer sensors.

Gyroscope	Accelerometer
2 integrated dual-axis, InvenSense 500 series	3 Axis Accelerometer, Freescale MMA7260Q
Measures angular rate	Sensitivity: 800 mV/g @ 1.5 g
Full scale range: ± 5000 deg/s	12 bit analogue digital converter (integer number)
Sensitivity: 2 mV/deg/s	Resolution: (1.5 g + 1.5 g)/($2^{12}$) ≈ 7.10 − 4 g/unit
12 bit ADC (integer number)	-

**Table 2:** CNN predefined parameters.

Parameter	Values
Learning Rate	0.001
Momentum Coefficient	0.9
No. of Feature Maps	32, 64
No. of Neurons in Fully Connected Layer	64
Batch Size	40
Epoch Number	19
Epoch Number	19

**Table 3:** MESF, SUM, and SVM performance using min and max pooling. Bold numbers represent the highest accuracy achieved for each subject using different methods.

	MESF		SUM		SVM	
	Max (%)	Min (%)	Max (%)	Min (%)	Max (%)	Min (%)
**Subject 1**	**95.56**	94.61	89.93	89.45	92.31	90.15
**Subject 2**	**100**	97.44	92.41	89.26	96.87	91.3
**Subject 3**	92.91	**94.26**	88.97	88.35	89.12	91.65
**Subject 4**	**99.35**	97.38	93.82	90.7	94.71	90.26
**Subject 5**	**95.76**	95.56	89.13	89.29	92.34	90.89
**Subject 6**	**97.27**	93.6	93.62	89.44	92.49	89.41
**Subject 7**	**96.15**	93.12	87.28	86.31	91.2	88.54
**Subject 8**	**98.93**	97.63	92.36	89.04	95.34	92.81
**Subject 9**	**94.65**	90.05	87.94	85.75	90.57	87.31
**Subject 10**	**100**	98.78	94.2	91.37	97.69	95.95
**Average**	**97.06**	95.24	90.96	88.89	93.27	90.83
